# Older persons’ narrations on falls and falling—Stories of courage and endurance

**DOI:** 10.3402/qhw.v10.26123

**Published:** 2015-01-08

**Authors:** Anne Clancy, Bjørg Balteskard, Bente Perander, Marianne Mahler

**Affiliations:** 1 Department of Health and Social Work, School of Nursing, Harstad University College, Harstad, Norway; 2 Department of Lifelong Learning and Philosophy, Aalborg University, Copenhagen, Denmark

**Keywords:** Fall prevention, Heidegger, Levinas, older persons, narratives, well-being, nursing care, health promotion

## Abstract

Fall related injuries in nursing homes have a major impact on the quality of life in later adulthood and there is a dearth of studies on falling and fall prevention from the older person's perspective. The aim of the study was to identify how older persons perceive falling, fall prevention, and fall accidents. Six in-depth interviews were carried out and a hermeneutic phenomenological method was used to describe and interpret the older persons’ accounts. Interpretations of Levinasian and Heidegarian philosophy related to dwelling and mobility helped cultivate important insights. Symbolic and physical environments are important for the participants’ well-being. The older persons in the study did not wish to dwell on the subject of falling and spoke of past and present coping strategies and the importance of staying on their feet. The women spoke about endurance in their daily lives. The men's narrations were more dramatic; they became animated when they spoke of their active past lives. As the scope of the study is small, these gender differences require further investigation. However, their stories give specific knowledge about the individual and their symbolic environmental circumstances and universal knowledge about the importance of integrating cultural environmental knowledge in health promotion and care work. Traditional fall prevention interventions are often risk oriented and based on generalized knowledge applied to particular cases. The findings indicate a need for contextual life-world knowledge and an understanding of fall prevention as a piece in a larger puzzle within a broader framework of culture, health, and well-being. Showing an interest in the older persons’ stories can help safeguard their integrity and promote their well-being. This can ignite a spark that kindles their desire to participate in meaningful exercises and activities.

Fall incidents are a threat to public health and a challenge in the care of older men and women. Injuries resulting from falls can shorten older persons’ lives (WHO, 2007), and expose their vulnerability. Despite recognition in the public health community that the health promotion concepts of settings, participation, and dialogue are necessary to achieve public health goals, a pathogenic risk focus continues to dominate the field of injury prevention. Traditional injury prevention is concerned with applying evidence-based generalized knowledge to particular cases. The assumption is often that appropriate physical and social environments can prevent fall accidents and promote well-being. The symbolic environment associated with spirituality, continuity, feeling safe, and carrying out meaningful activities is not in focus. The aim of this article is to expand on the traditional notion of fall prevention amongst older persons living in nursing homes by incorporating it within a broader framework of health promotion and well-being. Interviews are carried out with six older persons to get a greater understanding of what this broader approach can entail.

This study has a health promotion life-world approach to falls and falling. This approach acknowledges the complexities of health-related conditions to be more than the absence of disease and that well-being can offer a direction for caring (Galvin & Todres, 2011) and health promotion in older adults. It acknowledges that being attentive to the symbolic environment can support and promote a sense of well-being in older persons (Elo, Saarnio, & Isola, 2011).

## Injury prevention amongst older adults

According to the World Health Organization (WHO, 2007) older women injure themselves more than older men, but fall accidents amongst older men are more often fatal. Two-thirds of older persons needing nursing care in Norway live in institutions; where the welfare state is expected to look after those needing care, the figure is high in comparison with other countries (Daatland, Veenstra, & Lima, 2009). Frequency of falling is higher in residential care than among those living at home due to the ailments of the target group that lives in these facilities (Lord, Sherrington, Menz, & Close, 2007; Sadigh, Reimers, Andersson, & Laflamme, 2004).

Review studies in the Cochrane database 2009, 2010, and 2012 show that customized multifactorial fall prevention interventions have effect (Cameron et al., 2010; Gillespie et al., 2009). Multifactorial intervention programmes can reduce falls but not the risk of falling (Gillespie et al., 2012). The success of fall prevention programmes has also been problematized due to diversity in old age, and variations in culture and in living conditions (Gates, Fisher, Cooke, Carter, & Lamb, 2008; Jansson, 2007). Intrinsic factors have also been extensively studied (Vikman, Nordlund, Näslund, & Nyberg, 2011).

Public health professionals often focus on preventing falls from occurring. However, the majority of older persons are more concerned about the risk of losing or damaging their personal and social integrity; focus on health and independence can have a greater effect than traditional fall prevention programmes (Hughes et al., 2008). Increased focus on fall prevention and fall prevention programmes can induce worries and anxiety among the elderly (Mahler, 2012). There is a lack of qualitative studies that can give insight into the complexity of fall accidents and on how falls influence the lives of older people (Mahler, 2012; Mahler, Svensson, & Sarvimäki, 2011; Thomson & Hassenkamp, 2001).

## Health promotion and the concept of well-being

The purpose of this study is to create a broader understanding of fall prevention in the nursing home by incorporating the ideologies of health promotion and well-being to injury prevention practice. Boltz, Resnick, Capezuti, and Shuluk (2014) recommend a movement away from a focus on the absence of falls to one that emphasizes independence, self-direction, and the preservation and restoration of function in hospitalized older adults. A paradigm shift is also necessary in fall prevention approaches with older persons in municipal institutions. This study examines how a group of older North Norwegians living in nursing homes and an older person in an assisted care facility in the community narrate about how falling, fall accidents, and their prevention affect their lives.

Health promotion is not a discipline as such but a field of action that focuses on health not disease (Potvin & McQueen, 2007). It is grounded in a public health ethics of participation, responsibility, and equity (Tones & Tilford, 2001). A health promotion approach takes into consideration the well-being of each older person and groups of older persons within their culture and context. Health promotion acknowledges the importance of settings and of lay knowledge in the construction of evidence (Potvin & McQueen, 2007) as well as acknowledging the complexity of personhood, health, and illness (Galvin & Todres, 2013). The nursing home is not only a medical and health care institution but also a home for older persons within their local community. Narratives about falls can improve knowledge of how falls and fear of falling constrain the daily lives of older persons and point towards factors that can contribute to promoting their health and well-being. How a person perceives opportunities and constraints can determine how they face challenges (Bandura, 1982). The older person can have ailments and be dependent on nursing and medical care but they are people who have lived long lives and have not only accumulated ailments but also resources.

Understanding what gives the older person a good quality of life is important for their well-being. Sarvimäki (2006) discusses the concept of well-being and quality of life. The concept of well-being has traditionally either been divided into physical, social-mental, and spiritual well-being, or understood as a subjective experience of feeling good (Sarvimäki, 2006). A philosophical approach to well-being can give a deeper understanding of what it is. Heidegger criticizes the concept of human “being” as more than the sum of body, mind, and spirit (Sarvimäki, 2006). Heidegger is concerned with the truth of being and his philosophy can be termed a fundamental ontology (Peperzak, 1999).

Grounded in Heidegger's Philosophy of Being, Sarvimäki (2006) promotes an understanding of well-being in a holistic way by incorporating the life course as it unfolds in the ups and downs of day to day living. It acknowledges the importance of realizing one's potential as well as confronting anxiety and death. Well-being is contextual and well-being bound to places, which throughout life have been transformed into sites of great meaning, can reinforce individual and group identity (Rowles & Bernard, 2013).

Levinas’ fundamental philosophy adds a relational dimension to Heidegger's thesis on being as his philosophy is ethical and originates from the other, not the self (Levinas, 1969). *The other*, is a perspective that Heidegger does not delve deeply into (Peperzak, 1999).

The concepts of dwelling and mobility are relevant in the study of falls and falling in a nursing home setting where both mobility and “feeling at home” can be a challenge for older persons residing there. Heidegger and Levinas are concerned with the essence of *Being* (Peperzak, 1999) and both philosophies incorporate dwelling and mobility as the essence of well-being.

Todres and Galvin (2010) write about well-being as *dwelling–mobility* and refer to Heidegger's notion of “Gegnet” where being is not just a space for beings and things, but a wholeness, a home-like being in the world. Levinas (1969) meanwhile does not refer to being as a wholeness or totality but more as openness and movement as the totality of each human life on earth exists within an infinite ethics. Levinas (1969) writes about being at home in the world and how the intimacy of home is a given that precedes the useful. Levinas is concerned about relational movement, a movement involving the other, where the well-being of the other person is primal for my well-being. These philosophers can help us understand how existential *dwelling* and *mobility* are connected and how the deepest experience of well-being is related to both movement and stillness. The phenomena of dwelling and mobility are important, both literally and metaphorically for older persons living in a nursing home environment and for nursing staff who care for them there. These phenomena relate to both their physical and symbolic environments.

### Place and history as sites of meaning

This study was conducted in the north of Norway. The participants were born there between 1914 and 1927. To understand another person's world, researchers and practitioners need historical knowledge and a moral sense of place (Edvardsen, 1997, p. 141). Ageing cannot be understood as being solely a rigid predetermined “natural” phenomenon; it is influenced by food, nutrition, living conditions, and sex, as well as societal and cultural expectations and demands (Solem, 2008). The participants lived their lives in close contact with the elements. A sense of history is an important presence in the lives of older persons and permeates their environment (Elo, Saarnio, & Isola, 2011). The men had farmed, worked in the fishing industry, or built boats. The women catered to their families and had to endure the physical and mental strains of their husbands’ long absences at sea. The participants did not experience an abundance of wealth but due to rich natural resources, they could live off the land and sea and never went hungry. They continue to live in or near the area where they lived most of their lives.

## Material and methods

### Setting

The study was conducted in 2012–2013 at five different care facilities in two municipalities, one urban and one rural, in a region in Northern Norway. The region is located north of the Arctic Circle and has a rugged indented mountainous coastline. The winters are long with an abundance of snow, winter temperatures are often below freezing point, and snow covered or icy paths and roads are the norm. The winter climate and lack of sunlight can provide mobility challenges for the elderly necessitating that they spend more time indoors.

### Sample and ethical considerations

The criteria for inclusion were as follows: being aged 75 and older, living in a municipal institution (N) or other municipal care facility (C) in Northern Norway, and being able to adequately respond to the researchers’ questions. Over a period of 2 years, six older persons (aged between 75 and 98) were recruited by administrative staff at the care facilities. The staff were familiar with the inclusion criteria. All participants were given written and oral information, signed consent forms, and were informed that they could withdraw from the study at any time. The study was approved by the Norwegian social sciences data services and reviewed by the Regional Ethical Committee for the North of Norway. Table I provides an overview of the sample and settings.

**Table I:** Overview of the sample, interviews, and settings for the field observations.

Two urban and two rural nursing homes (N)^a^ One urban intermediary care facility (C)	

Length of interviews: between 40 min and 1 h Length of observations between 45 min and 4 h (six visits, total 15 h)	

Interviewees (gender and age)	Researchers focus during interviews

Eva 87 (N)	Information from the researcher on the project
Martin 89 (N)	“Tell me a little about yourself”
Nils 98 (N)	Time spent in the nursing home
Vera 75 (N)	Experience of falling, fall events
Anna 82 (C)	Feeling safe, or insecure
Hans 82 (N)	“Situations where you feel a sense of security” “Situations where you feel most at risk” Use of safety equipment

Participant names are fictitious.

a Observations were carried out at one rural and one urban nursing home.

### Data collection and analysis

Data was collected using open interviews that were recorded verbatim and a research diary was written. The interviewer spoke about the focus of the study and then let the participants narrate their experiences (Table I). The persons were aged between 75 and 98, their strength was taken into account, and the interviews lasted no more than 1 h. The first author drafted the article. The first, second, and third authors carried out the interviews and all four authors participated in thematization of the findings and subsequent discussion. A series of six observations was carried out at two nursing homes between 900 and 1500 h over a period of 6 weeks. The researchers spent time in the nursing home sitting rooms, kitchens, and corridors. The observations, though limited, can be termed as ethnographic as they involved immersion and participation in order to understand a way of life (Creswell, 2007; Patton, 2002). Participation involved being with the residents, speaking to them, and sharing meals with them. The observations provide supplementary data and are not the main focus of the article. This article focuses on the interview data. Van Manen's (1997) selective highlighting approach to text analysis is used. This hermeneutic phenomenological method of researching lived experience is both descriptive and interpretive in its form. It comprises collecting experiential material in the form of a text, then highlighting sentences, phrases, or words that can bring forth the essence of each participant's lived experience. The philosophies of Heidegger and Levinas mediate with the authors throughout the analysis, not to frame or constrain our understanding, but to inspire and recover meaning from the interview texts. Van Manen (2014) reminds us that phenomenology is a philosophical project that requires attentive creativity and that philosophical insight cultivates inspiration.

## Findings

The findings from the descriptive interpretative analysis of the interviews are presented under the following themes: Stories of courage and endurance; Meaningful activities; and “I am not afraid of falling or dying” (Table II). Supportive data from the observations are included where relevant.

**Table II:** Highlighted themes and examples from the interpretive process.

Highlighted sentences and phrases	Interpretive process	Main themes
Fell at home, not much to tell (F 82).		
Falling: don't think too much about it (F 87) (F 75). M 98 said that he had never fallen. I fell and broke off my hip bone (M 82).	Not an interesting subject to dwell on. The fall incident in itself is not important but for some of the men showing their strength and sharing the drama of its aftermath is.	
I remember once I fell at the table, I became paralysed and couldn't get up and had to crawl over to the bed (…) the whole floor was covered in blood (M 82).		
I was out for the count banged my head on the wall and then on the floor. I have banged my head so many times, I‘m surprised it's still stuck on (M 89).	An interesting subject with elements of drama	
I remember once when I was working on an artic seal hunting ship. We started to cut with the welding torch, there was smoke everywhere (…). Normally we cut from the outside, but this time we had no choice and had to work from the inside. There was no ventilation. I was in there in the thick of cutting. My nephew was with me, he asked me if I was “gone in the head.” The room was black with smoke; there were no floorboards so I had to jump from beam to beam. F*** hell, when I think of what I have been through and endured. (…) I was a welder, a mechanic. I was everything (…). I was a plumber. I had to keep going (M82).	The men became optimistic when they spoke about their past. Telling stories about their former lives gave the men a chance to show their strength and endurance.	*Stories of courage and endurance*
I am finished, completely finished, I am stuck here at the nursing home. The most important thing is to stay on my feet (…) but when I just lie here its ok, but when I get up especially in the morning it's (dizziness) terrible (M89).		
I used to work; I remember I cut 5 acres of grass with a scythe one summer. 40–50 women came to the island during the summer; they dried and salted the cod, laid it out on the rocks to dry (M89).		
The nurse dropped me (…). It was because of the roller walker that I fell (F 82). M 98 Pointed to his foot, that's the problem, look at that. The dizziness is terrible (M 89).		
I believe that I will get better day by day, you have to be optimistic, can't be negative.	Externalizing the problem	*Meaningful activities*
I don't want to use the walker, I want to use my legs (F 82).		
I am an old lady so as long as I'm up and about and can manage I am satisfied. I have my ailments, heart and back, but I manage (F 87).	The women adapted to their circumstances	
I try to be independent but I don't find the roller walker easy to use. I have cushioned that handle and I try to practice (F 82).	Being optimistic and dealing with their ailments.	
When I am about to get out of bed I know if I am man enough to walk on my own, I don't feel that I am taking a chance. I decide on the spur of the moment and I see that he (the roller walker) is over there and say “You stay, I go.” It's when I feel well enough. If I fall, I fall, I am not afraid of falling or dying (M 82).		*I am not afraid of falling or dying*
I used to go fishing with my brothers, we survived storms at sea. (Illustrated with his arms how he kept his balance on the fishing boat) (M 98).

F=female. M=male. Numbers illustrate age in years.

### Stories of courage and endurance

The observations and interviews showed that the nursing home residents lived with many ailments. Their form varied from day to day. They said that they felt safe and well looked after, even though they would have preferred to live at home had they been well enough. They knew that this was not a realistic option. The residents had reduced mobility due to strokes, pulmonary conditions, and other ailments. They spoke of symptoms, such as pain, dizziness, shortness of breath, walking difficulties, and bladder problems.

Falling as such was not an interesting subject to dwell on for the participants. Their answers on the subject were often short, as illustrated in the following quotes: “Fell at home not much to tell” (Anna, 82). Eva, 87, and Vera, 75, said that they did not think much about falling; Nils, 98, said that he had never fallen. The interviewees were more interested in talking about their present ailments or their past life as active citizens.

Even though they did not dwell on falls, two of the men spoke in a dramatic away about fall incidents:

I remember once I fell at the table, I became paralysed and couldn't get up, had to crawl over to the bed (…) the whole floor was covered in blood. (Hans, 82)

I was out for the count, banged my head on the wall and then on the floor. I have banged my head so many times, I‘m surprised it's still stuck on. (Martin, 89)

These older men with many ailments became animated and transformed when they spoke about their past lives. A mostly bedridden oxygen-dependent man with episodic bouts of severe pain became a multitasking genius when given the opportunity to narrate about his life. Illustrated by the following quote (Hans, 82):

I remember once when I was working on an artic seal hunting ship. We started to cut with the welding torch, there was smoke everywhere (…). Normally we cut from the outside, but this time we had no choice and had to work from the inside. There was no ventilation. I was in there in the thick of cutting. My nephew was with me, he asked me if I was “gone in the head.” The room was black with smoke; there were no floorboards so I had to jump from beam to beam. F*** hell, when I think of what I have been through and endured. (…) I was a welder, a mechanic I was everything (…). I was a plumber. I had to keep going.

Nils, 98, who had severe mobility problems and sat in a wheelchair, became a mountain climber and seafarer when he spoke about his past life. He spoke of hunting trips, slippery decks, and rough seas and, hands outstretched, demonstrated to the researcher how he kept his balance at sea and avoided falling. Martin, 89, spoke of an active life and about his strength and endurance as a young man. As he said:

I used to work, I remember I cut five acres of grass with a scythe one summer. 40–50 women came to the island during the summer, they dried and salted the cod and laid it out on the rocks to dry.

Recent falls the participants described occurred mainly indoors and were associated with getting in and out of bed or standing up. Extrinsic factors were blamed for falls. As Anna, 82 said, “the nurse dropped me” and when describing another fall said, “It was because of the roller walker that I fell.” Ailing body parts were also blamed. Two patients pointed to a paralysed limb as if it didn't belong to them. Nils, 98, pointed to his foot and said, “that's the problem look at that.” As Martin, 89, said (pointing to his legs), “that there is the problem.” He also spoke of “the dizziness” and said:

I am finished, completely finished; I am stuck here at the nursing home. The most important thing is to stay on my feet (…) but when I just lie here its ok, but when I get up especially in the morning the dizziness is terrible.

### Meaningful activities

The patients agreed that they could not survive without nursing care. Observations confirmed both this and their dependency on safety equipment and walking aids. Roller walkers were not merely walking aids they were regarded by the informants as vital appendages required to get through the day. Martin, 89, spoke of the roller walker as his friend, and Hans, 82, personalized the roller walker and spoke directly to it.

Keeping their independence and being active was important. Vera, 75, said, “I can't walk without my roller walker, I have to have it.” Anna, 82, said:

I try to be independent but I don't find the roller walker easy to use. I have cushioned that handle and I try to practice. I don't want to use the walker; I want to use my legs.

Eva, 87 was often out for walks even in the wintertime. She said, When the roads are icy I use studded boots, they are a bit too heavy. I would like to try other models. I prefer the crampons. I use walking poles as well to increase my stability.

The men spoke about activities at the nursing home. Martin, 89, said that the most important thing was to stay on his feet. Martin spoke about being bored and the possibility of participation in nursing home activities. He said:

As far as I know they (the staff) get old people to knit, I could do that if I could get my fingers around it. I can't walk about but could do something with my arms.

Researcher: What about mending fishing nets?

No, but I could play the accordion, I used to do that before, wonder where it is now?

The observations illustrated passivity in the nursing home sitting rooms. The staff were there and catered to individual needs. The patients mostly sat there, television on. Nobody seemed to be watching it.

The sitting room has the character of a waiting room, the staff are present they wander back and forth, other than the noise from the TV and the odd call for help there is silence. (Field notes)

### “I am not afraid of falling or dying …”

The interviewees had to adapt to their situation. The men were more pessimistic about the future, the women endured. They were all realistic about their situation. Martin, 89 said: “If it's slippery outside I just don't go out.” Hans, 82, said:

When I am about to get out of bed I know if I am man enough to walk on my own, I don't feel that I am taking a chance. I decide on the spur of the moment and I see that he (the roller walker) is over there and say “You stay, I go.” It's when I feel well enough. If I fall, I fall, I am not afraid of falling or dying.

The women struggled on. Illustrated by the following comments:

I believe that I will get better day by day, you have to be optimistic, can't be negative. (Anna, 82)

I am an old lady so as long as I‘m up and about and can manage I am satisfied. I have my ailments, heart and back, but I manage. (Eva, 87)

The narratives illustrate what is important in the older people's lives. Falls are not an interesting subject to dwell on. They look back, and acknowledge their dependency but they also struggle on and see the importance of staying on their feet. Their stories are tales of courage and endurance.

## Interpretation and discussion

### Well-being in falling and ageing

When the former fishermen, boat builders, mechanics, and leaders had the chance to talk to a researcher, they did not wish to dwell on episodic falls that had no bearing on who they were or wanted to be seen as. Reluctance to talk about falls has been noted in former studies (Mahler & Sarvimäki, 2010). This is understandable as a fall happens in the space of a split second and can be an occurrence that the older person does not recall. A fall is an event beyond their control that can be humiliating, and difficult to understand and verbalize.

The men became animated when they spoke about their past lives as active citizens and when they shared certain fall stories that had elements of drama. Well-being can be related to a sense of adventure (Galvin & Todres, 2011). Individuals who share life stories often use typical story patterns that are generally in use in a culture (Horsdal, 2002). Stories about the elements and about fishing and hunting are an important part of life in the north of Norway (Edvardsen, 1997). Through their stories, the men could share their life history and show who they were through their physical strength and prowess.

Autobiographical memory enabled the participants to travel in time. Recalling memories of strength and vitality was a form of mental time travel. Horsdal (2002) suggests a connection between mental time travel and bodily movement in space reminding us that mental time travel is crucial for physical journeys. This important connection needs to be investigated further. It seemed as if the men's imaginative journeys into the past gave them a new lease of life; an energy that reminded them more of their abilities than their disabilities. Galvin and Todres (2011) write that well-being can be experienced in mood and in the experience of personal identity; a sense of movement that can give access to new horizons and possibilities. The 98-year-old resident, with arms outstretched, actively demonstrating how he balanced on a boat as a younger man, facilitated vitality and a meaningful bodily response to the memory — an illustration of balance that gave more meaning than a balance exercise programme that might not have any bearing on his life, past or present. Generalized exercise programmes can be successful in preventing falls and in promoting an idea of well-being. However, generalized well-being is not necessarily authentic well-being for each individual (Todres & Galvin, 2010).

Galvin and Todres (2011) mention future orientation in their descriptions of well-being experiences. Imaginative journeys into the past can also be important for hope and a felt sense of possibilities. Recollecting past experiences, and knowing that they can share their stories with attentive nursing staff, can give the residents something to look forward to, and, in that sense, create an orientation towards the future. Well-being can be about small things; it is anything that offers an invitation into a welcoming future (Galvin & Todres, 2011). Narratives can be disempowering if they are not taken up by a listener, and being listened to is a moral act that can contribute to well-being (Medeiros, 2014). Thinking with stories does not mean that we work on the narratives but allow the narratives to work on us (Frank, 1995). This can ensure that the nurse does not interrupt and use her expert knowledge on fall prevention to change focus but instead holds back and allows the story to develop. The older person's narrative can hold a wealth of valuable information, which can in turn be incorporated into safety promotion practice.

Sarvimäki (2006) encourages health researchers to seek alternative ways of describing well-being. Our re-description of well-being in safety promotion illustrates the importance of recalling memories and the sense experience of mental time travel. Memories can provide comfort (Elo, Saarnio, & Isola, 2011). Remembering how to balance on a boat or on a beam in a shipyard can help frail bodies remember and remind them of their strength. Narrating these episodes can give the older person a sense of well-being and of place and continuity. A trip from the bed to the table can require the same prowess as balancing on a boat in a storm in the North Sea or the exertion of harvesting five acres of grass with a scythe. A familiar place can offer itself as a starting point for literal and metaphorical journeys (Galvin & Todres, 2011). Forgetting their present plight by telling stories from their past can also give a sense of identity and the mental strength necessary to participate in nursing home activities.

Keeping their independence was important for the participants. Boredom and pessimism were evident in their stories but they were orientated towards the future. The women soldiered on and had a “grin and bear it” attitude. All had strategies and aids they used to “stay on their feet” and prevent falls. The older persons showed continued strength and they perceived struggling with illnesses to be a part of life to be endured.

The residents’ acceptance and orientation towards the future can be indicative of well-being, though not well-being at its fullest. “At homeness” is characterized by acceptance and rootedness (Galvin & Todres, 2011). It can be questioned if the participants felt at home in the institution. Rootedness can be interpreted as passivity and their “grin and bear it” attitude interpreted as a form of forced adjustment and resignation. True well-being incorporates both “at homeness” and adventure, a sense of rootedness and flow, movement as well as stillness (Galvin & Todres, 2011). The observations showed passivity in the nursing home sitting rooms. This can be due to the necessity of a calm atmosphere that caters for very ill residents. It does, however, not seem to be a stimulating home environment for all the residents considering their past lives and interests. Picking up threads from their past life can facilitate meaningful exercises and activities and show that health professionals have more to offer patients than knitting.

It is necessary to stimulate the symbolic environment, adjust the physical environment, and ask “What sense of both home and adventure can be offered to this particular person in order to promote safety and well-being?” Well-being as a mooded sense is anything that motivates a connection with a person's desires and motivates them into recognizing that life is worth living (Galvin & Todres, 2011).

The participants in this study are concerned about being mobile and used phrases such as “staying on my feet,” “being up and about,” and “keep going.” These phrases can be interpreted as metaphors for activity and participation. The participants know that they are “on their last legs,” and see the importance of staying on their feet. Lying down is associated with illness and the difficulty and drama of getting up. The residents battle with diseases and ailments, they know that they are at death's door, each fall can be fatal, falling down can be their last fall and be synonymous with dying. As mentioned in the introduction, Sarvimäki (2006) promotes an understanding of well-being that unfolds in “the ups and downs” of day to day living. Health and life are up, sickness and death are down (Lakoff & Johnson, 1980) and when the older persons use orientational metaphors for activity and participation they show their orientation towards the future and see themselves as “still up and running.” These are what Lakoff and Johnson (1980) would call structural metaphors, metaphors that shape perceptions and actions otherwise unnoticed.

Finding meaning and purpose in strenuous activities can encourage activity and participation. Studies have shown the stigma and humiliation of falling and the importance of fall stories in creating a sense of coherence in daily life (Mahler & Sarvimäki, 2010). The participants’ use of metaphors for activity and participation can be indicative of their need to focus on living, not dying; give them a sense of continuity; and allow them to “re-emerge” as resourceful courageous persons. The older person's stories showed life courage in their continued endurance. Life courage is defined by Pahuus (1995), the Danish philosopher, as an “active energy.” Older persons may wish to withdraw from life, but they also want and need to participate in life and in meaningful activities (Balteskard, Storli, & Martinsen, 2013; Moe, 2013).

The older persons spoke of illnesses, balance difficulties, weak body parts, and inadequate safety equipment as extrinsic factors. Isolating the problem as something external seemed to be a helpful strategy in taking control over their lives. Stories told about our lives can make a big difference to how we experience our lives (Roesler, 2006). This strategy seemed to put them in control and promoted their sense of identity and well-being. Creating a necessary distance to a problem is a way of dealing with it; an external factor is a challenge to be overcome (Polkinghorne, 1996).

## Well-being in safety promotion

The deepest experience of well-being is a unity of dwelling and mobility (Galvin & Todres, 2011). Safety promotion connected with well-being incorporates attentiveness to symbolic environments not just physical ones; environments that can house both imaginative and literal activities and journeys. This approach necessitates that nursing carers are attentive to the older person's narratives, stories that have seemingly no bearing on fall prevention or their present situation. Stories told can promote the tellers well-being and remind the listener of who these persons are. Nurses and other carers are important listeners that can be changed by the stories they hear as they acquire a store of cultural knowledge (Frank, 1995; Medeiros, 2014), a knowledge that can subsequently provide a more open approach to injury prevention practice.

The philosophers, Heidegger and Levinas were presented in the introduction. Interpretations of Heideggerian philosophy have helped create meaning from the participants’ stories. Levinas’ ethics of responsibility is especially relevant for health professionals. There is danger in a narrow injury prevention approach that focus on the protection of vulnerable body parts, such as osteoporotic hips or fragile wrists becomes the main concern. The older person's disabilities and dependency can prevent nurses and other staff from seeing who these persons are. Levinas (1969) insists that moral responsibility entails a relational movement towards the other and attentiveness to their differences. Openness to older person's stories about what is important in their lives can counter dominant master injury prevention narratives that have a generalized pathogenic risk focus. Attentiveness to stories can help reclaim the identity of individuals and move the nurses so that both nurses and patients can be, in Levinasian terms, “borne beyond the given” (Levinas, Peperzak, Critchley, & Bernasconi, 1996, p. 34). Levinas’ philosophy sharpens the concept of *difference* and not *sameness* as a point of departure for health promotion nursing activities and warns against the pitfalls of assuming that other persons or groups of persons have a common identity.

## Methodological reflections

Although the number of women and men participating in the study is small, their narratives provide both specific and universal knowledge: Specific knowledge about the individual and their symbolic environmental circumstances and universal knowledge about the importance of integrating cultural environmental knowledge in health promotion and care work. With the life-world philosophy as a point of departure, Van Manen (1997, 2014) helped us see the importance of life-world descriptions and the necessity of creative probing in interpreting and understanding human experiences. The authors see the necessity of further studies concerning falling, environmental understanding, and health promotion. This study will be followed up by an interview study with nursing staff on falls and fall prevention in nursing homes.

## Conclusion

The scope of this study is small and the gender differences illustrated in the study cannot be generalized. The study set out to reveal how six older persons experience falls and falling. Phenomenological philosophy provided inspirational insight. Heidegger prompted us to be attentive to modes of being in the world, whereas Levinas reminds nurses to understand and address the otherness of each older person. Probing the residents’ experiences revealed a deeper meaning. The experience of falls and falling that presents itself in the participants’ stories reveals life courage and endurance and is more about getting up, staying up, and moving on than falling down.

The findings support the need for contextual knowledge and an individualized open approach to safety promotion and fall prevention in institutions, and that an emphasis on well-being can offer a direction for safety promotion in older adults. These findings are relevant for all public health professionals involved in fall prevention who run the risk of objectifying the individual older person as having preconceived generalized needs.

The findings are an important reminder that nurses, therapists, and other carers should regard fall-risk management as a piece of a larger puzzle. Putting together the pieces of the puzzle requires a process of understanding that is created by attentiveness to the individual older person and acknowledging their resources and strategies. The findings are relevant in all areas of care. Showing an interest in the older persons’ lives can ignite the spark that kindles the older person's life spirit, no matter their ailments. Attentive staff can help boost the older person's well-being, safeguard their integrity, and promote real participation in appropriate fall prevention strategies. Generalized knowledge on injury prevention is important but it cannot stand alone. Listening attentively to the older person's narrations about the ups and downs of daily life can provide a deep well of knowledge that can promote an understanding of both symbolic and physical environments.
